# Identification of candidate genes associated with feather follicle traits in chickens

**DOI:** 10.1093/jas/skag291

**Published:** 2026-11-05

**Authors:** Changbin Zhao, Dajian Li, Ming Pan, Ze Zhang, Xiquan Zhang, Dexiang Zhang, Hongmei Li

**Affiliations:** State Key Laboratory for Conservation and Utilization of Subtropical Agro-Bioresources, Lingnan Guangdong Laboratory of Agriculture, College of Animal Science, South China Agricultural University, Guangzhou 510642, P.R. China; State Key Laboratory for Conservation and Utilization of Subtropical Agro-Bioresources, Lingnan Guangdong Laboratory of Agriculture, College of Animal Science, South China Agricultural University, Guangzhou 510642, P.R. China; Animal Husbandry Technology Extension Section, Zhanjiang Livestock Technology Extension Station, Zhanjiang 524094, P.R. China; State Key Laboratory for Conservation and Utilization of Subtropical Agro-Bioresources, Lingnan Guangdong Laboratory of Agriculture, College of Animal Science, South China Agricultural University, Guangzhou 510642, P.R. China; State Key Laboratory for Conservation and Utilization of Subtropical Agro-Bioresources, Lingnan Guangdong Laboratory of Agriculture, College of Animal Science, South China Agricultural University, Guangzhou 510642, P.R. China; State Key Laboratory for Conservation and Utilization of Subtropical Agro-Bioresources, Lingnan Guangdong Laboratory of Agriculture, College of Animal Science, South China Agricultural University, Guangzhou 510642, P.R. China; State Key Laboratory for Conservation and Utilization of Subtropical Agro-Bioresources, Lingnan Guangdong Laboratory of Agriculture, College of Animal Science, South China Agricultural University, Guangzhou 510642, P.R. China; State Key Laboratory for Conservation and Utilization of Subtropical Agro-Bioresources, Lingnan Guangdong Laboratory of Agriculture, College of Animal Science, South China Agricultural University, Guangzhou 510642, P.R. China

**Keywords:** yellow-feathered chicken, feather follicles, transcriptome, *HOXB3*, single nucleotide polymorphisms

## Abstract

The size and density of feather follicles are important appearance quality traits for slaughtered broilers. In China, consumers generally prefer broilers with smooth skin and fine follicles, yet the genetic and molecular basis of these traits remains poorly understood. In this study, we characterized feather and feather follicle traits in 589 yellow-feathered chickens at 100 d of age and investigated candidate genes and SNPs associated with feather follicle size. Skin microscopy combined with image analysis provided a practical approach for quantifying follicle size. The size of feather follicles, the weight, length, and diameter of feathers were significantly higher in males than in females (*P* < 0.05), indicating clear sexual dimorphism. Feather follicle traits also showed greater variation than feather traits, which may limit the efficiency of direct selection. Correlation analysis showed a higher genetic association between dorsal feather length and follicle size of dorsopelvic tract, suggesting the potential for indirect selection of feather follicle size. RNA sequencing of large and small feather follicle tissues identified 1,273 differentially expressed genes. These differentially expressed genes were primarily enriched in the Wnt, MAPK, calcium, and TGF-β signaling pathways. Further analysis identified several *HOX* family genes, including *HOXC10*, *HOXD11*, *HOXB3*, and *HOXD12*, which may play crucial roles in feather follicle development. The tissue expression profile indicates that the *HOXB3* gene is highly expressed in skin tissue. Polymorphism analysis of *HOXB3* identified three SNPs significantly associated with feather follicle size (*P* < 0.05). These findings provide a practical method for follicle-size measurement and identify candidate pathways, genes, and SNP markers for the genetic improvement of feather follicle traits in chickens.

## Teaser

By integrating skin microscopy, quantitative genetics, transcriptome analysis, and SNPs association analysis, this study identified measurable indicators, biological pathways, and candidate genetic markers associated with chicken feather follicle traits.

## Introduction

Yellow-feathered broilers are an important indigenous chicken type in China and are valued for their tender meat and distinctive flavor. In recent years, measures related to avian influenza control and the COVID-19 pandemic have accelerated the transition from live-bird marketing to centralized slaughter and the sale of chilled or frozen poultry in China (Pan et al. 2020; Li et al. 2022). As a result, carcass appearance has become increasingly important to consumers. Feather follicle density, size, and diameter are key components of skin appearance, and Chinese consumers generally prefer carcasses with smooth skin and small, inconspicuous feather follicles.

Feather follicles support feather formation, growth, pigmentation, and regeneration (Ji et al. 2023), indicating a close relationship between feathers and feather follicles. Jenni et al. (2020) have revealed a significant correlation between the size of feather follicles (feather root cross-sectional area) and the length and structure of feathers across various bird species (Jenni et al. 2020). In recent years, follicular traits have already been studied in several species due to their appearance attribute and close association with feather and hair development, such as chickens (Chen et al. 2020; Xie et al. 2020), ducks (Chen et al. 2017; Yang et al. 2018), geese (Hu et al. 2020; Song et al. 2022), and sheep (Zhao et al. 2021; He et al. 2022). Feather follicle traits are complex quantitative traits that are influenced by multiple genes and their interactions (Ji et al. 2023). However, the genetic parameters and molecular basis of feather follicle variation in yellow-feathered chickens remain poorly understood. Clarifying these factors is important for identifying candidate genes and improving feather follicle traits through breeding.

RNA sequencing (RNA-seq) enables high-throughput quantification of transcript abundance and the identification of differentially expressed genes (DEGs) between biological groups. Wang et al. (2024) compared chickens with high and low feather follicle densities and identified several candidate genes, including forkhead box M1 (*FOXM1*), G2 and S phase expressed 1 (*GTSE1*), maternal embryonic leucine zipper kinase (*MELK*), and cyclin-dependent kinase 1 (*CDK1*) (Wang et al. 2024). Using microarray analysis of chicken back and thigh skin, Ji et al. (2021) identified DEGs, including insulin-like growth factor 1 (*IGF1*), fibroblast growth factor 9 (*FGF9*), and homeobox B6 (*HOXB6*), that were enriched in extracellular matrix (ECM) receptor interaction, focal adhesion, and Wnt signaling pathways (Ji et al. 2021). They also identified a WNT3A SNP, g.255361C > A, that was significantly associated with feather follicle density. A SNP is a DNA sequence variant involving a change at a single nucleotide position (Vignal et al. 2002). Because of their abundance, genome-wide distribution, and genetic stability, SNPs are widely used in livestock breeding and disease research (O’Brien et al. 1999). Sun et al. (2019) conducted a genome-wide association study (GWAS) of contour feather density in chickens and identified 146 significant SNP markers and several candidate genes, including succinate-CoA ligase ADP-forming subunit beta (*SUCLA2*), DnaJ heat shock protein family (Hsp40) member C15 (*DNAJC15*), dehydrogenase/reductase 12 (*DHRS12*), motilin receptor (*MLNR*) and retinoblastoma 1 (*RB1*) (Sun et al. 2019). However, most previous studies have focused on feather follicle density, whereas the genetic and molecular basis of follicle size remains poorly understood. Investigating variation in feather follicle size may therefore provide candidate markers for improving carcass appearance in yellow-feathered chickens.

In this study, we first evaluated the use of skin microscopy and image analysis to quantify feather follicle size. We then estimated genetic parameters and examined relationships between feather follicle and feather traits to identify potential indirect indicators of follicle size. RNA-seq was used to identify DEGs and signaling pathways associated with follicle size, and the tissue-expression patterns of selected candidate genes were further examined. Finally, we analyzed *HOXB3* polymorphisms and their associations with feather follicle size. This study aimed to characterize the genetic basis of feather follicle traits and identify candidate genes and genetic variants associated with follicle size in yellow-feathered chickens.

## Material and methods

### Experimental animals, trait measurement, and calculation

All animal experiments were performed according to the protocols approved by the South China Agricultural University Institutional Animal Care and Use Committee (Approval number: SCAU#0017). All animal procedures followed the regulations and guidelines established by this committee and minimized the suffering of animals.

The study examined a test population of 589 late-feathering yellow-feathered (Qingyuan partridge) chickens, including 293 males and 296 females, sourced from Guangdong Wenshi Southern Poultry Breeding Co., Ltd (Guangdong, China). Each chicken was moved to the research brooder after assigning wing numbers and was vaccinated against various diseases including Marek disease, Newcastle disease and bronchitis at 1-d-old. After 4 wk, they were transitioned to a ground-level flat rearing setup. All chickens were kept in the same environment until 100 d of age and slaughtered. They had free access to feed and water, and the feed nutrient levels are shown in Table S1. The test population was transported to the XinXing Poultry Co., Ltd. slaughterhouse for centralized processing on the evening prior to the assay. Post-slaughter, carcasses were refrigerated at 4°C and then allowed to equilibrate to room temperature for 30 min before feather follicle analysis. All individual measurements were conducted within 4 h of slaughter. Feather sampling occurred the day preceding slaughter, feathers were stored in parafilm bags after plucking, each marked with a wing number. All feathers are of the third generation, exhibiting complete morphology and development. To eliminate the influence of bilateral asymmetry of the secondary flights feather on the results (Aparicio 1998), all individuals had the secondary flights feather collected from the left side. Subsequent feather trait analyses were carried out in the Poultry Genetics Breeding and Reproduction Laboratory at the College of Animal Science, South China Agricultural University. All assay indices were measured as described below:

Feather follicle size on back (BFFS): Feather follicles on the dorsopelvic tract (Pass 1995) of chickens were imaged using a PC35 skin microscope (CK, Cologne, Germany). The relative area of the feather follicles in the images was quantified with ImageJ software. A minimum of five follicles was assessed to calculate the mean follicle size for each individual. Measurements were taken twice per individual, and the relative deviation between the two readings was maintained below 10%. The average of these two measurements was used to represent the relative size of the feather follicles on the individual’s back. For ease of calculation, the unit of measurement was converted from pixels (px) to kilopixels (kpx).

Feather follicle size index on back (BFFSI): The ratio of dorsopelvic tract feather follicle size to body weight (px/g) was established as an index to negate the influence of body weight on feather follicle dimensions.

Density of feather follicle (DFF): The number of feather follicles within 2 × 2 cm^2^ of skin at the dorsopelvic tract of chickens was counted.

Length of flight feather (LFF): The length of the left fifth mature primary flight feather was extracted from the chickens, and it was measured from the tip of the feather to the end of calamus using steel ruler, unit: cm.

Weight of flight feather (WFF): The weight of the fifth mature primary flight feather from the left wing of the chickens was measured using an analytical balance, unit: g.

Length of flight feather calamus (LFFC): The length of the left fifth mature primary flight feather was extracted from the chickens, and it was measured from the junction between the feather shaft and the calamus to the end of the calamus using steel ruler, unit: cm.

Diameter of flight feather calamus (DFFC): The length of the left fifth mature primary flight feather was extracted from the chickens, the diameter at the midpoint of the primary flight feather calamus was measured using vernier calipers, unit: mm.

Length of contour feather on back (LCF): Three mature dorsal contour feathers located at the dorsopelvic tract were extracted, and the length from the tip of each feather to the end of calamus was measured using a steel ruler. These measurements were then averaged, unit: cm.

Length of contour feather calamus on back (LCFC): The length from the junction of the three mature dorsal contour feathers shaft and the calamus to the end of the calamus at the dorsopelvic tract of chickens was measured using vernier calipers, and the average value was recorded as the measurement, unit: mm.

Diameter of contour feather calamus on back (DCFC): The diameters at the midpoint of the three mature dorsal contour feathers calamus at the dorsopelvic tract of chickens were measured using vernier calipers and then averaged, unit: mm.

Weight of contour feather on back (WCF): Five to six mature dorsal contour feathers located at the dorsopelvic tract of chickens were extracted and weighed collectively using an analytical balance. The ratio of the total weight to the number of feathers determined the weight of a single back feather for that individual, unit: mg.

Body weight (BW): The live weight of the chickens was measured, and feeding was halted for 12 h prior to the measurement, unit: g.

The description of feather and feather follicle traits was shown in Figure 1.

**Figure 1:**
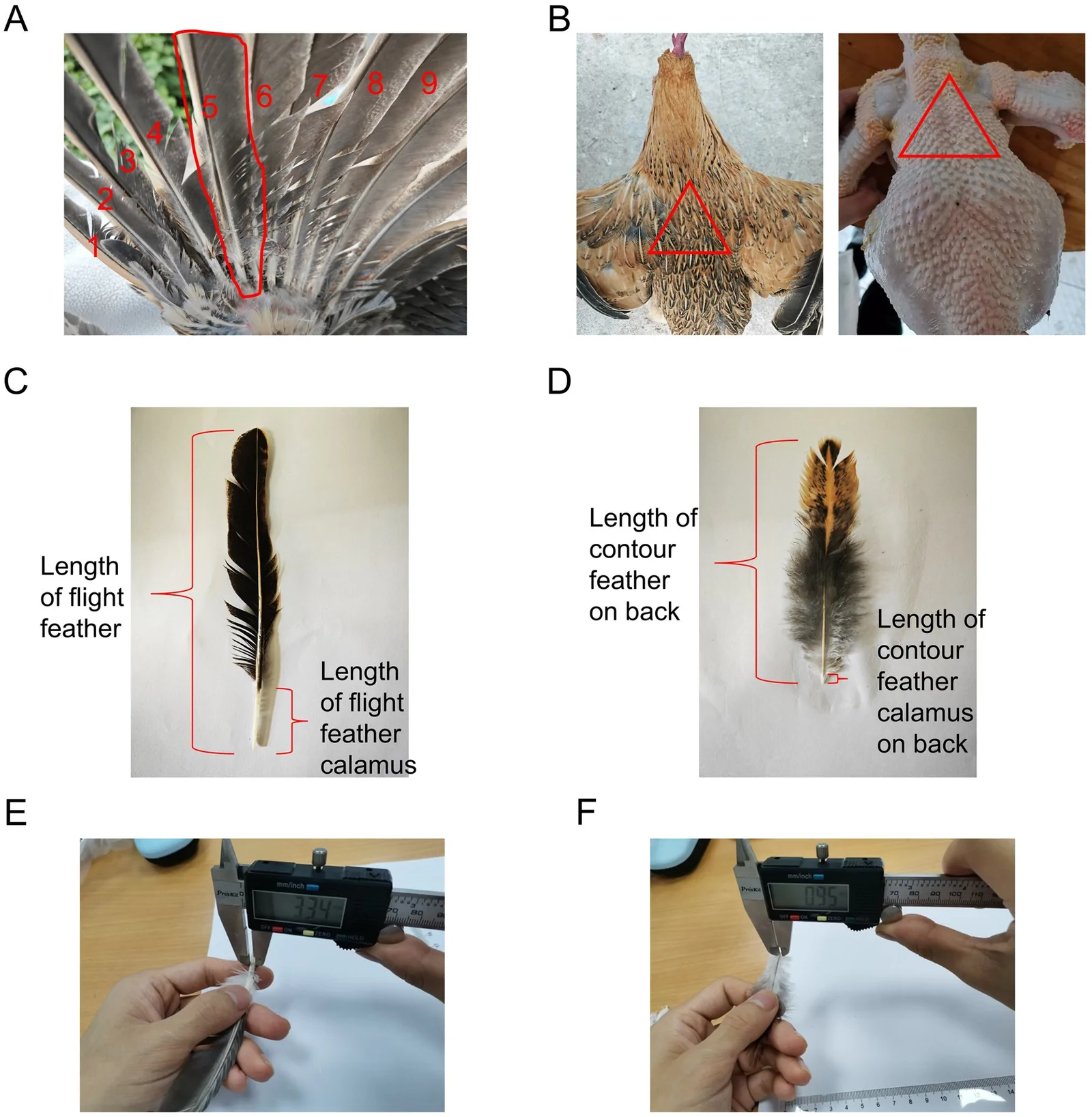
Measurement of feather and feather follicle traits in yellow-feathered chickens. A) Primary flight feathers were counted inward from the outermost primary on the left wing. The fifth primary flight feather selected for trait measurement is outlined. B) Sampling region of the dorsal contour feathers and feather follicles in the dorsopelvic tract. C) Representative photograph illustrating the measurement of flight feather length and flight feather calamus length. D) Representative photograph illustrating the measurement of dorsal contour feather length and contour feather calamus length. E) Representative photograph illustrating the measurement of flight feather calamus diameter using vernier calipers. F) Representative photograph illustrating the measurement of contour feather calamus diameter using vernier calipers.

In addition, four 100-d-old yellow-feathered chickens with similar weights were selected. The liver, heart, spleen, lung, kidney, pectoral muscle, leg muscle, subcutaneous fat, abdominal fat, skin, cerebellum, and bone marrow were collected to characterize the tissue expression profiles of *HOXB3* mRNA.

### DNA extraction

A total of 332 chickens were randomly selected from the population of 589 yellow-feathered chickens for genotyping. Genomic DNA was extracted from their blood samples using the E.Z.N.A. SQ Blood DNA Kit (Omega, Norcross, Georgia) following the manufacturer’s protocol. The concentration of DNA samples and the optical density (OD) value at 260/280 were measured using a Nanodrop 2000c spectrophotometer (Thermo Fisher, Waltham, Massachusetts). The samples were then stored at −80°C for later use.

### Polymerase chain reaction (PCR)

Primers based on the *HOXB3* gene sequence provided by Ensembl (accession number: ENSGALG00010023428) were designed using the NCBI Primer-BLAST (Primer designing tool (https://www.ncbi.nlm.nih.gov/tools/primer-blast/)). The primer information is presented in Table S2. PCR was conducted in a total volume of 30 μL, including 1 μL of DNA template, 15 μL of 2 × Green Taq Mix (Vazyme, Nanjing, China), 1.2 μL each of forward and reverse primers, and 11.6 μL of ddH_2_O. The PCR cycling conditions consisted of an initial denaturation at 95°C for 3 min, followed by 34 cycles of denaturation at 95°C for 15 s, annealing at 57°C for 15 s, and extension at 72°C for 90 s, with a final extension at 72°C for 5 min. The PCR products were then analyzed by electrophoresis using a 1.5% agarose gel. The PCR products were sequenced using the Sanger sequencing method by Sangon Biotech Co. Ltd. (Shanghai, China) to detect gene mutations.

### RNA sequencing and bioinformatics analysis

Five 100-d-old yellow-feathered chickens from the same population were selected for the study. Feather follicle samples, large feather follicles (LF) from the secondary flights feather follicle and small feather follicles (SF) from the dorsopelvic tract, were collected from each chicken. Mean follicle size was greater in the LF group than in the SF group (253.24 ± 12.48 vs. 174.54 ± 8.55, *P *< 0.001, *n* = 5, sex = female, unit: kpx). The samples were immediately placed into cryovials, snap-frozen in liquid nitrogen, and subsequently stored in an −80°C freezer. Trizol reagent (Invitrogen, Waltham, Massachusetts) was used to extract total RNA from samples according to the manufacturer’s instructions. Agilent 2100 Bioanalyzer (Agilent, Santa Clara, California) was used to analyze the purity and integrity of RNA, and high-quality RNA samples (2.0 > OD260/280 > 1.8, OD260/230 > 2.0) were selected for library construction. The steps of library construction are as follows: the mRNA with polyA tail is enriched by oligo (dT) magnetic beads, and then the resulting mRNA is randomly interrupted by divalent cations in the fragmentation buffer. The first strand of cDNA was synthesized using fragmented mRNA as the template and random oligonucleotides as primers in the M-MuLV reverse transcriptase system, then the RNA strand was degraded using RNaseH, and the second strand of cDNA was synthesized from dNTPs in DNA polymerase I system. After the purified double-stranded cDNA was end repaired, a-tailed, and connected to the sequencing adaptor, the cDNA screened with AMPure XP beads was amplified by PCR, and the PCR product was purified again with AMPure XP beads, resulting in the final library. After detection by Agilent 2100 Bioanalyzer, qualified libraries were sequenced on the Illumina Novaseq (San Diego, California) platform and 150 bp paired end reads were generated.

Then, nine transcriptome sample datasets were obtained, including four from the LF group (one sample failed sequencing) and five from the SF group. To ensure the quality and reliability of data analysis, it is necessary to filter the original data. This mainly involves removing reads with adapters, reads containing N (N indicates undetermined base information), and low-quality reads (reads with Qphred ≤5 that had more than 50% of the entire read length in bases). Post-quality filtering, reads were aligned to the reference genome GRCg7b (https://www.ncbi.nlm.nih.gov/datasets/genome/GCF_016 699485.2/) via HISAT2 software. Gene expression levels were normalized as FPKM (fragments per kilobase of transcript per million mapped reads). Genes with FPKM > 0.1 were considered expressed genes. Sample correlation analysis and principal component analysis (PCA) were then performed based on the normalized expression matrix. Differential expression analysis employed normalized read counts, utilizing DESeq2 with parameters set to |log_2_ fold change| ≥ 1 and *P*-adjust < 0.05. Volcano plots were generated using the R package ggplot2. For heatmap visualization, the expression values of each DEG were standardized across samples using row-wise Z-score scaling, and hierarchical clustering of genes and samples was performed using Euclidean distance and the complete-linkage method with the R package pheatmap. Following DEGs analysis, genes were categorized based on genome annotation information. Enrichment analyses for Kyoto Encyclopedia of Genes and Genomes (KEGG, http://www.kegg.jp/) and Gene Ontology Functional Enrichment Analysis (GO, http://www.geneontology.org/) were conducted using clusterprofiler, with significance determined using hypergeometric distribution and a threshold *P*-value < 0.05. Protein–protein interaction information for the DEGs was retrieved from the STRING database, with Gallus gallus selected as the organism and the minimum required interaction score set to 0.4 (medium confidence). The resulting interaction network was imported into Cytoscape. A subnetwork containing differentially expressed HOX proteins and their directly interacting proteins was extracted for visualization. Pearson’s correlation analysis was performed between the log_2_ fold changes obtained by RNA-seq and quantitative real-time PCR (qRT-PCR) for the four validated *HOX* genes.

### RNA extraction, cDNA synthesis, and qRT-PCR

qRT-PCR validation was performed using five independent biological samples per group. RNA was extracted from tissues using RNAiso reagent (Takara, Kusatsu, Japan) following the manufacturer’s protocol. The concentration of RNA samples was determined, and the optical density (OD) value at 260/280 was measured using a Nanodrop 2000c spectrophotometer (Thermo Fisher). The RNA samples were stored at −80°C for future use. For qRT-PCR, cDNA synthesis was performed using the MonScript™RTIII All-in-One Mix with dsDNase (Mona, Wuhan, China). qRT-PCR was performed using the ChamQ Universal SYBR qPCR Master Mix (Vazym) on a Bio-Rad CFX96 Real-Time Detection instrument (Bio-Rad, Hercules, California) according to the manufacturer’s instructions. The reaction consisted of an initial denaturation at 95°C for 3 min, followed by denaturation at 95°C for 10 s and annealing at 60°C for 30 s, repeating for a total of 40 cycles. The dissociation curve was analyzed by gradually increasing the temperature from 65 to 95°C. Relative gene expression was determined by performing qRT-PCR in duplicate for each reaction, with *GAPDH* serving as the internal control. The primers used in qRT-PCR are provided in Table S3.

### Statistics and analysis

Genotyping of 14 SNPs in the *HOXB3* gene was performed in 332 randomly selected yellow-feathered chickens using SeqMan software. Outliers in the data for each trait were removed based on the principle of μ ± 3σ. Phenotypic data were assessed for normal distribution using the R package “pastecs,” and descriptive statistics, including mean, standard deviation, coefficient of variation, maximum, and minimum values, were computed for each trait. Trait distributions were visualized using the R package ggplot2. Allele and genotype frequencies, observed heterozygosity (Ho), expected heterozygosity (He), polymorphism information content (PIC), the effective number of alleles (Ne), and Hardy-Weinberg equilibrium *P*-values were calculated using PopGene32 software. To compare allele distributions across different chicken populations, publicly available genotype and allele frequency data for five *HOXB3* SNPs in Chinese and Korean chicken populations were obtained from Ensembl population genetics data derived from EVA study PRJEB44919. Sample sizes were calculated from the reported genotype counts. The genetic diversity indices were calculated as follows:


$$\mathrm{Ho}\;=\;N_{H}/N$$

$$\mathrm{He}\;=\;1\;-\;\sum {p_{i}}^{²}$$

$$\mathrm{Ne}\;=\;1/\sum {p_{i}}^{²}$$

$$\mathrm{PIC}\;=\;1\;-\sum {p_{i}}^{²}\;-\;\sum \sum 2{p_{i}}^{²}{p_{j}}^{²}$$

*N*_H_ is the number of heterozygous individuals, *N* is the total number of individuals, and *p*_i_ and *p*_j_ are the frequencies of the *i*th and *j*th alleles, respectively.

The heritability of each trait, genetic correlations, and phenotypic correlations between traits were estimated using ASReml software based on pedigree information from 589 yellow-feathered chickens. Heritability was estimated using a single-trait animal model, and genetic and phenotypic correlations between pairs of traits were estimated using a two-trait animal model. The animal model for each trait was:


y = Xb + Za + e

*y* is the vector of phenotypic observations, *b* is the vector of fixed effects including sex, *X* is the incidence matrix for fixed effects, *a* is the vector of individual additive genetic effects, *Z* is the incidence matrix for additive genetic effects, and e is the vector of residuals. Data are presented as the mean ± standard deviation (SD). For two-group comparisons, statistical significance was assessed using a two-tailed Student’s *t*-test.

Association analysis between SNPs and phenotypic traits was performed using mixed linear models in SPSS Statistics software. The analysis model is as follows:


$$Y_{\mathrm{ijlm}}\;=\;\mu \;+\;G_{i}\;+\;S_{j}\;+\;f_{l}\;+\;e_{\mathrm{ijlm}}$$

*Y*_ijlm_ is the observed value, *μ* is the overall average, *G*_i_ is the fixed effect of the genotype, *S*_j_ is the fixed effect of sex, *f*_l_ is the random effect of the family, and *e*_ijlm_ is the random error term. Multiple comparisons among genotypes were adjusted using the Bonferroni method.

## Results

### Assessment of feather follicle size using skin microscopy

We evaluated the use of standardized skin microscopy combined with image analysis to compare feather follicle size. Images of 2 × 2 cm skin areas were captured using the same magnification and imaging settings, and follicle areas were quantified. Representative images from chickens with LF and SF are shown in Figure 2A and B, respectively. The measured follicle areas were 253,588 pixels for the large-follicle sample and 183,522 pixels for the small-follicle sample. Thus, the measured area of the large follicles was approximately 38.2% greater than that of the small follicles. These results indicate that skin microscopy combined with ImageJ provides a practical approach for the relative comparison of feather follicle size under standardized imaging conditions.

**Figure 2:**
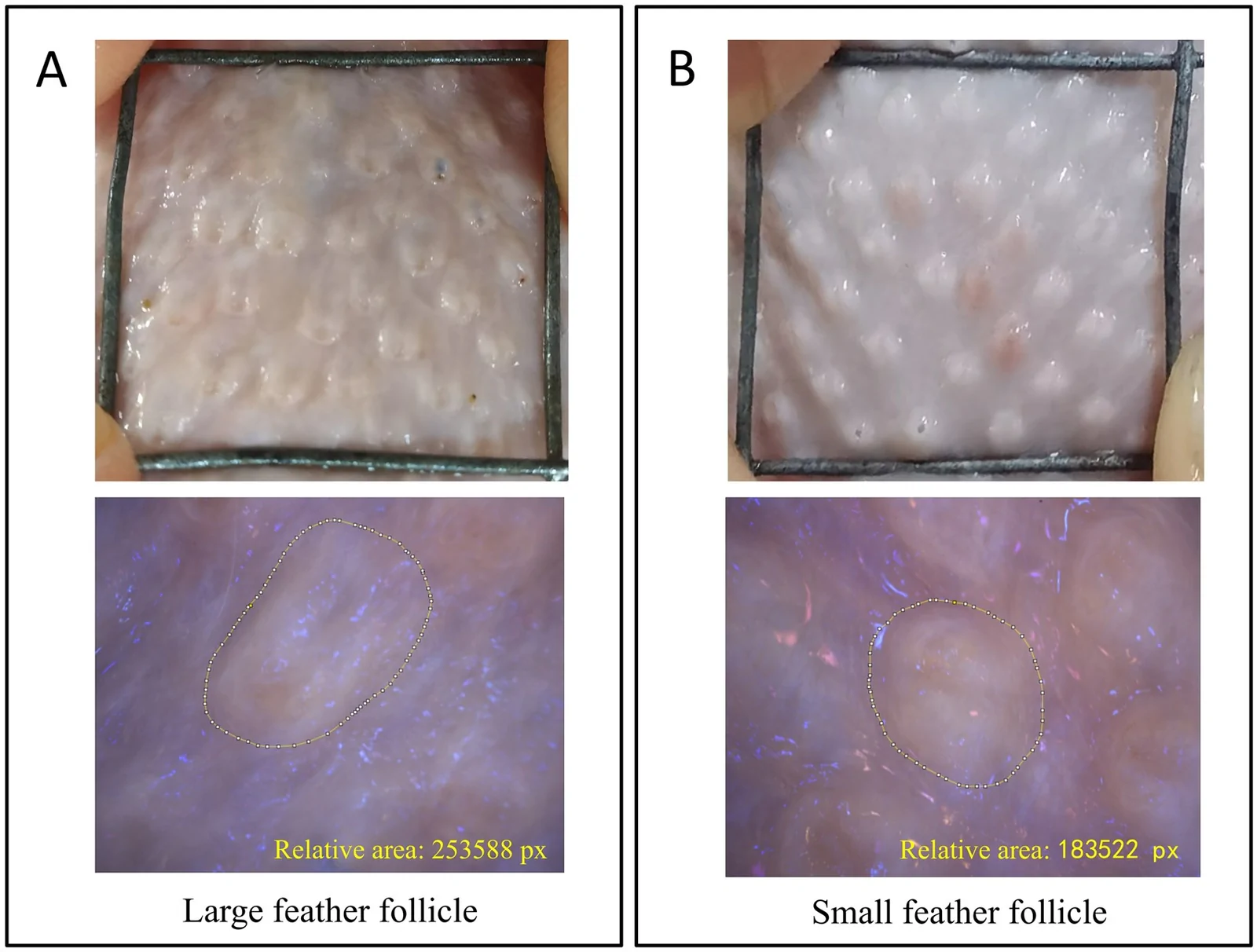
The actual and relative sizes of yellow-feathered chicken feather follicles. A) The relative area between 2 × 2 cm^2^ photographs of individuals with large feather follicles and their corresponding measurements after magnification using a skin microscope, unit: px. (B) The relative area between 2 × 2 cm^2^ photographs of individuals with small feather follicles and their measurements after magnification using a skin microscope, unit: px.

### Sexual dimorphism and phenotypic variation in feather follicle and feather traits in chickens

The results of the phenotypic analysis of feather traits (LFF, WFF, LFFC, DFFC, LCF, WCF, LCFC, DCFC), feather follicle traits (BFFS, BFFSI, DFF), and body weight in 589 yellow-feathered chickens are summarized in Table 1. All feather trait values were greater in males than in females (*P *< 0.01). The feather follicle trait BFFS showed significantly larger values in males (214.04 kpx) compared to females (185.24 kpx) (*P *< 0.001), while BFFSI and DFF did not show significant differences between males and females (*P *> 0.05). Additionally, males had significantly higher body weight than females (*P *< 0.001).

**Table 1:** Descriptive statistics of feather follicle and feather traits in yellow-feathered chickens.

Traits	Sex	Number	Mean ± SD^1^	Coefficient of variation, %	Minimum	Maximum	*P*-value^2^
**LFF, cm**	Male	293	17.94 ± 0.73^A^	4.07	16	20	<0.001
	Female	296	16.29 ± 0.74^B^	4.54	14.4	18.4	
**WFF, mg**	Male	293	277.40 ± 30.50^A^	10.99	188.90	365.30	<0.001
	Female	296	203.20 ± 17.80^B^	8.76	169.80	249.10	
**LFFC, cm**	Male	293	3.53 ± 0.29^A^	8.22	2.8	4.4	<0.001
	Female	296	2.97 ± 0.21^B^	7.07	2.4	3.6	
**DFFC, mm**	Male	293	3.05 ± 0.18^A^	5.90	2.58	3.58	<0.001
	Female	296	2.80 ± 0.14^B^	5.00	2.48	3.18	
**LCF, cm**	Male	293	7.10 ± 0.42^A^	5.92	5.87	8.27	<0.001
	Female	296	6.40 ± 0.34^B^	5.31	5.43	7.43	
**WCF, mg**	Male	293	21.80 ± 3.40^A^	15.60	11.80	30.70	<0.001
	Female	296	19.20 ± 3.00^B^	15.62	11.10	27.60	
**LCFC, mm**	Male	293	3.13 ± 0.23^a^	7.35	2.57	3.81	0.0012
	Female	296	3.07 ± 0.18^b^	5.86	2.56	3.53	
**DCFC, mm**	Male	293	0.89 ± 0.08^A^	8.99	0.68	1.10	<0.001
	Female	296	0.82 ± 0.05^B^	6.10	0.69	0.96	
**BFFS, kpx**	Male	293	214.04 ± 30.25^A^	14.13	140.14	301.46	<0.001
	Female	296	185.24 ± 31.90^B^	17.22	105.90	272.44	
**BFFSI, px/g**	Male	293	125.80 ± 21.06	16.74	77.19	180.20	0.8029
	Female	296	126.33 ± 26.48	20.96	71.55	194.90	
**DFF**	Male	293	31.47 ± 3.67	11.66	22	40	0.5205
	Female	296	31.67 ± 3.46	10.92	22	40	
**BW, g**	Male	293	1,710.18 ± 197.64^A^	11.56	1,150	2,285	<0.001
	Female	296	1,486.80 ± 162.75^B^	10.95	1,065	1,950	

1 Within each trait, means with different lowercase letters differ significantly at *P *< 0.05, whereas means with different uppercase letters differ significantly at *P *< 0.001. Means without letters are not significantly different.

2 *P*-value: Student’s *t* test was used to evaluate the significance of the difference between unpaired samples and the means.

Moreover, the variation coefficients for feather traits WFF and WCF were relatively large, with values of 10.99% for males in WFF and 15.60% for all in WCF. Similarly, the coefficients of variation for feather follicle traits BFFS, BFFSI, and DFF were all above 10%, with values of 14.13%, 17.22%, 16.74%, 20.96%, 11.66%, and 10.92% for the corresponding categories. The coefficient of variation for body weight was also relatively high, at 11.56% for males and 10.95% for females. The distribution of intraindividual coefficients of variation for BFFS and the overall interindividual coefficient of variation are shown in Figure 3. The mean intraindividual and interindividual coefficients of variation were 12.4% and 17.4%, respectively. These results indicate considerable variation in feather follicle size both within and among individuals, which may reduce the precision of direct phenotypic selection.

**Figure 3:**
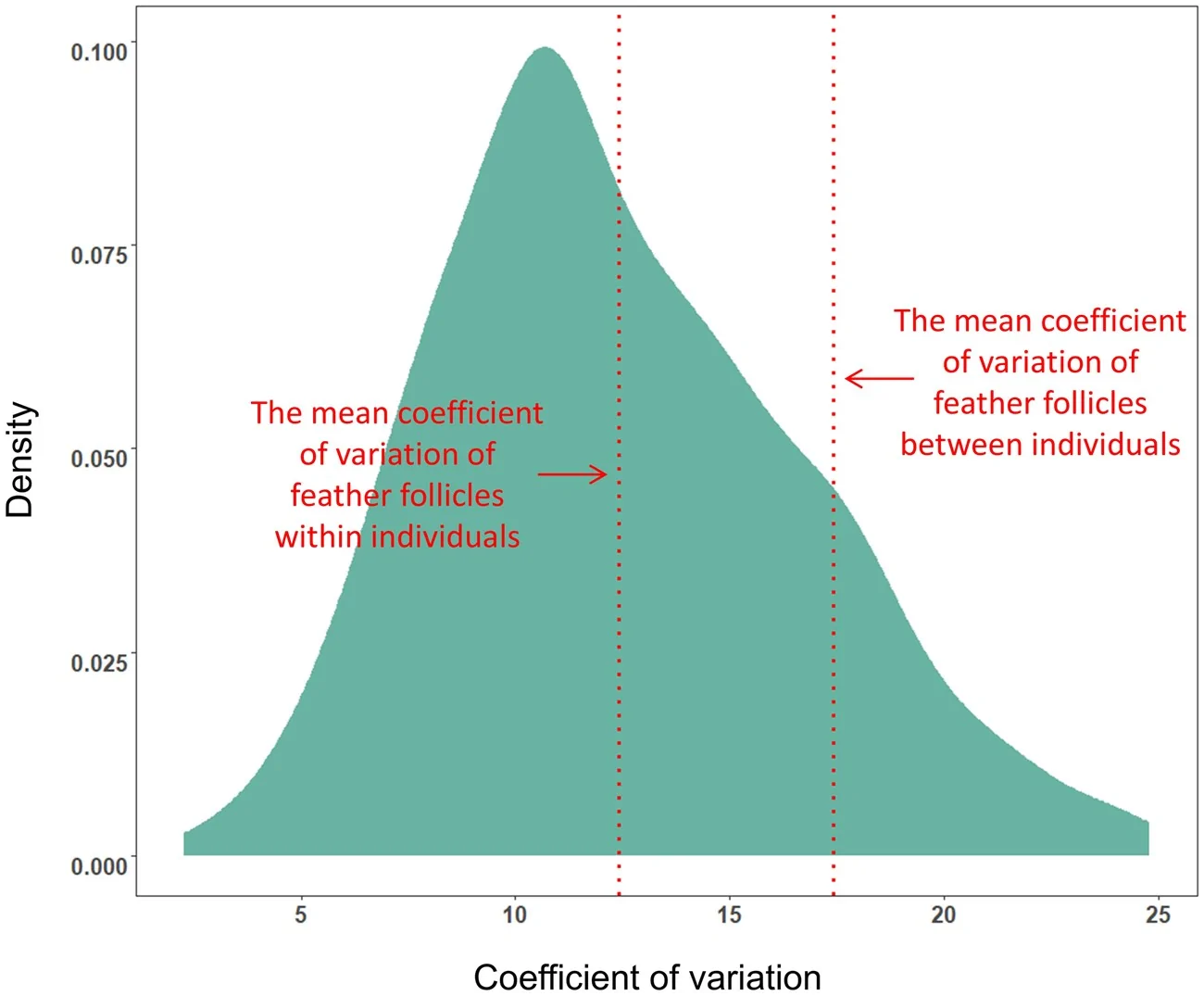
The density plot of intraindividual and interindividual coefficients of variation for chicken feather follicle size in yellow-feathered chickens (*n* = 589).

### Heritability and correlations among feather and feather follicle traits

To investigate the association between feather follicle traits and feather traits in chickens, we conducted genetic and phenotypic correlation analyses and estimated the heritability of each trait using data from 589 yellow-feathered chickens, with the results presented in Table 2. LFFC, LCF, WCF, BFFS, and BFFSI showed low heritability, with estimates of 0.13, 0.10, 0.15, 0.20, and 0.18, respectively. LCFC and BW exhibited moderate heritability, with estimates of 0.39 and 0.30, respectively. LFF, WFF, DFFC, DCFC, and DFF showed medium to high heritability, with estimates of 0.45, 0.44, 0.42, 0.53, and 0.44, respectively.

**Table 2:** Heritability (on the diagonal, standard errors in brackets), phenotypic correlations (above the diagonal), and genetic correlations (below the diagonal) between traits in yellow-feathered chickens (*n* = 589).

Traits	LFF	WFF	LFFC	DFFC	LCF	WCF	LCFC	DCFC	BFFS	BFFSI	DFF	BW
**LFF**	0.45 (0.13)	0.84**	0.61**	0.66**	0.60**	0.37**	0.12**	0.36**	0.38**	−0.02	−0.05	0.51**
**WFF**	0.77**	0.44 (0.13)	0.55**	0.83**	0.62**	0.44**	0.12**	0.50**	0.42**	−0.07	−0.08	0.62**
**LFFC**	0.18	0.31	0.13 (0.08)	0.28**	0.44**	0.22**	0.22**	0.29**	0.33**	−0.01	−0.02	0.42**
**DFFC**	0.44*	0.84**	−0.06	0.42 (0.12)	0.53**	0.45**	0.09	0.51**	0.31**	−0.11*	−0.11*	0.54**
**LCF**	0.16	0.26	−0.21	0.14	0.10 (0.07)	0.73**	0.19**	0.57**	0.32**	−0.11*	−0.15**	0.54**
**WCF**	0.21	0.48	−0.10	0.58*	0.44	0.15 (0.07)	0.05	0.63**	0.41**	−0.23**	−0.20**	0.52**
**LCFC**	−0.28	−0.03	−0.05	−0.05	−0.07	−0.10	0.39 (0.12)	0.20**	0.07	0	0.04	0.07
**DCFC**	−0.08	0.17	0.34	0.34	0.30	0.69**	0.36	0.53 (0.12)	0.28**	−0.03	−0.07	0.39**
**BFFS**	0.06	0.18	0	0	−0.34	0.45	−0.15	0.17	0.20 (0.09)	0.72**	−0.14**	0.24**
**BFFSI**	−0.01	−0.12	−0.33	−0.33	−0.49	−0.04	−0.03	0.13	0.73**	0.18 (0.09)	0.07	−0.48**
**DFF**	−0.11	−0.01	0.09	0.09	−0.40	−0.24	0.28	0.28	−0.28	0.35	0.44 (0.14)	−0.28**
**BW**	0.16	0.38	0.44	0.44*	0.48	0.66**	−0.11	0.05	−0.02	−0.68**	−0.71**	0.30 (0.10)

* Indicates a significant correlation at the level of 0.05 (2-tailed).

** Indicates a significant correlation at the level of 0.01 (2-tailed).

The genetic correlation results revealed a highly significant positive correlation (*P *< 0.01) between WFF and LFF among feather traits (correlation coefficient = 0.77). Significant positive correlations were also found between DFFC and LFF, as well as between WFF and WCF (*P *< 0.05). BW showed significant correlations with DFFC and WCF (*P *< 0.05). Additionally, there was a highly significant negative correlation (*P *< 0.01) between BW and DFF (correlation coefficient = −0.71). Furthermore, BFFS exhibited a positive, though non-significant (*P *> 0.05), correlation with both WFF and WCF, with correlation coefficients of 0.18 and 0.45, respectively.

The phenotypic correlation results revealed significant positive correlations (*P *< 0.05) among feather traits, except for LCFC, which did not significantly correlate with DFFC and WCF (*P *> 0.05). The correlation coefficients ranged from 0.12 to 0.84 among these traits. BW showed significant correlations with other traits (*P *< 0.05), except for LCFC. Similarly, BFFS exhibited significant positive correlations (*P *< 0.05) with other feather traits, except for LCFC, with correlation coefficients ranging from 0.28 to 0.42. BFFSI was negatively correlated with DFFC, LCF, and WCF (*P *< 0.05), whereas its correlations with the remaining feather traits were not significant.

### Differential expression analysis between the SF and LF groups

To investigate the molecular mechanism underlying the formation of feather follicle size, we conducted transcriptome sequencing on LF tissues from the secondary flight feather follicles and SF tissues from the dorsopelvic tract, including four LF samples and five SF samples. The total reads ranged from 40,704,554 to 45,450,472 per sample. The mean total reads were 42,617,753 ± 1,547,833 in the LF group and 43,303,214 ± 1,740,494 in the SF group. The percentage of reads aligned to unique locations in the reference genome ranged from 82.86% to 90.29% across samples, and over 16,500 expressed genes were detected (Table S4). PCA was performed based on the FPKM values of each sample to visualize the overall transcriptomic patterns. The PCA plot showed a group-related distribution pattern (Figure 4A). Using the thresholds of |log_2_ fold change| ≥1 and adjusted *P*-value < 0.05, we identified 1,273 DEGs between the LF and SF groups (Figure 4B). Among these DEGs, 434 genes were upregulated and 839 genes were downregulated in the LF group relative to the SF group. Subsequently, we generated a heatmap of the DEGs between the LF and SF groups (Figure 4C).

**Figure 4:**
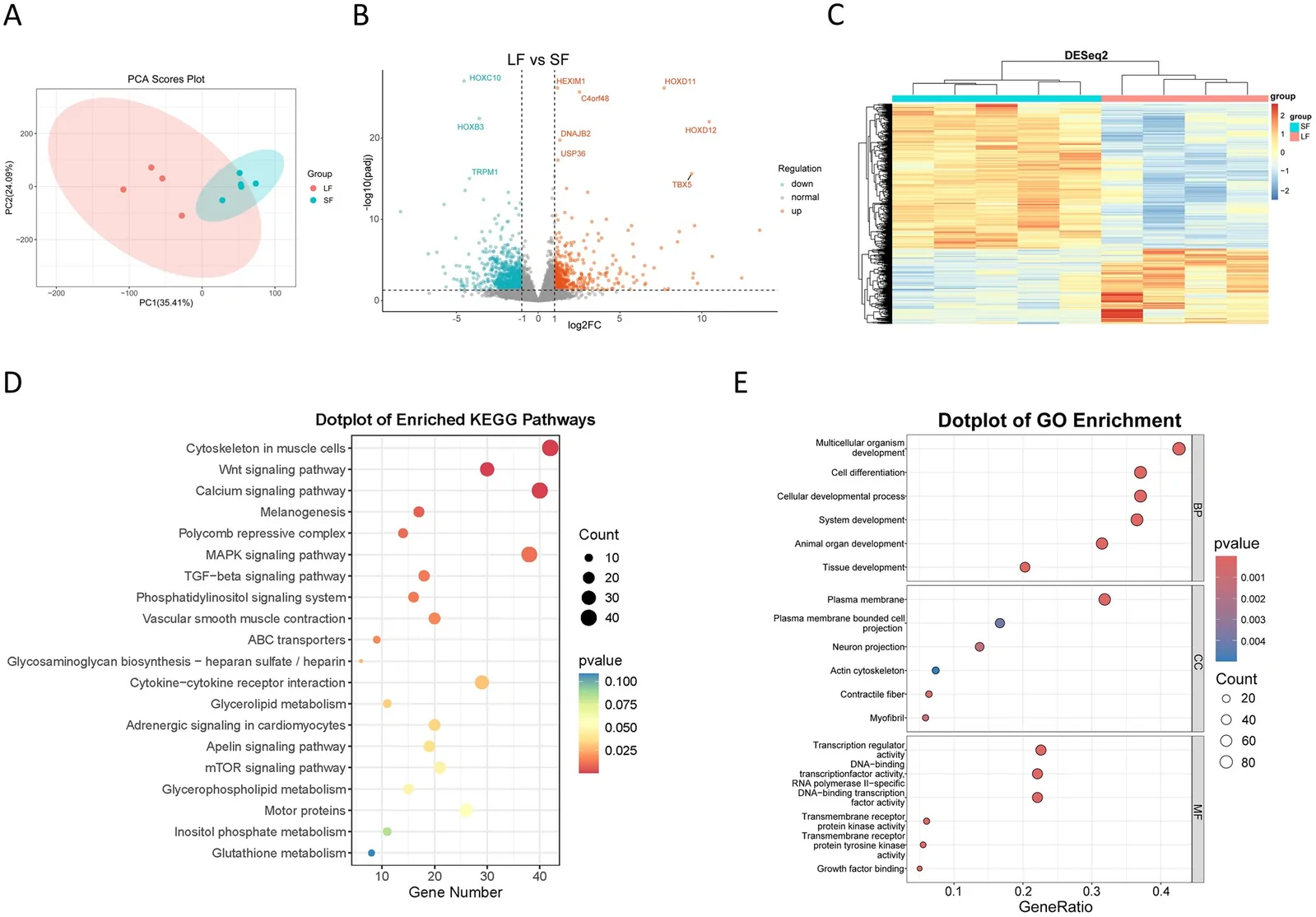
Differential expression analysis was conducted between the SF and LF groups in yellow-feathered chickens. A) The principal component analysis of RNA-seq samples (LF group, *n* = 4; SF group, *n* = 5). B) Volcano plot of DEGs between the LF and SF groups. The vertical dashed lines represent log_2_ fold-change thresholds of −1 and 1, and the horizontal dashed line represents an adjusted *P*-value threshold of 0.05. C) The heat map obtained by clustering analysis of DEGs. D) KEGG enrichment analysis of DEGs between SF and LF. E) GO enrichment analysis of DEGs between the LF and SF groups, including biological process (BP), cellular component (CC), and molecular function (MF) categories.

To explore the biological functions of the 1,273 DEGs, we conducted KEGG and GO enrichment analyses. The KEGG analysis revealed enrichment of DEGs in pathways such as the Wnt signaling pathway, calcium signaling pathway, MAPK signaling pathway, and TGF-β signaling pathway (Figure 4D). GO enrichment analysis was performed for three categories: biological process (BP), cellular component (CC), and molecular function (MF). In the BP category, DEGs were enriched in cellular developmental process, cell differentiation, tissue development, and system development. In the CC category, DEGs were enriched in plasma membrane, plasma membrane-bounded cell projection, neuron projection, and actin cytoskeleton. In the MF category, DEGs were enriched in transcription regulator activity, DNA-binding transcription factor activity, and transmembrane receptor protein kinase activity (Figure 4E). These findings suggest the potential involvement of these biological processes in feather follicle development.

### Protein interaction network construction and validation of DEGs

To further analyze the DEGs, we selected the top 15 most significantly upregulated and downregulated genes based on *P*-values and displayed them in a heat map (Figure 5A). Notably, several of the highly significant DEGs, including *HOXC10*, *HOXD11*, *HOXB3*, and *HOXD12*, belong to the homeobox transcription factor family. Detailed information about these specific genes can be found in Table S5. To explore the role of *HOX* family genes among DEGs, we constructed a protein–protein interaction network for the differentially expressed *HOX* genes (Figure 5B). Members of the *HOXB* and *HOXC* gene families were predominantly downregulated, whereas members of the *HOXA* and *HOXD* gene families were predominantly upregulated in the LF group relative to the SF group. We further examined the expression patterns of these differentially expressed *HOX* genes across tissues and cell types using publicly available ChickenGTEx transcriptomic data (Guan et al. 2025). *HOXB3*, *HOXC10*, *HOXD11*, and *HOXD12* showed relatively high expression levels in skin and embryonic tissues (Figure S1, see online supplementary material for a color version of this figure), suggesting that these genes may be associated with skin and embryonic development.

**Figure 5:**
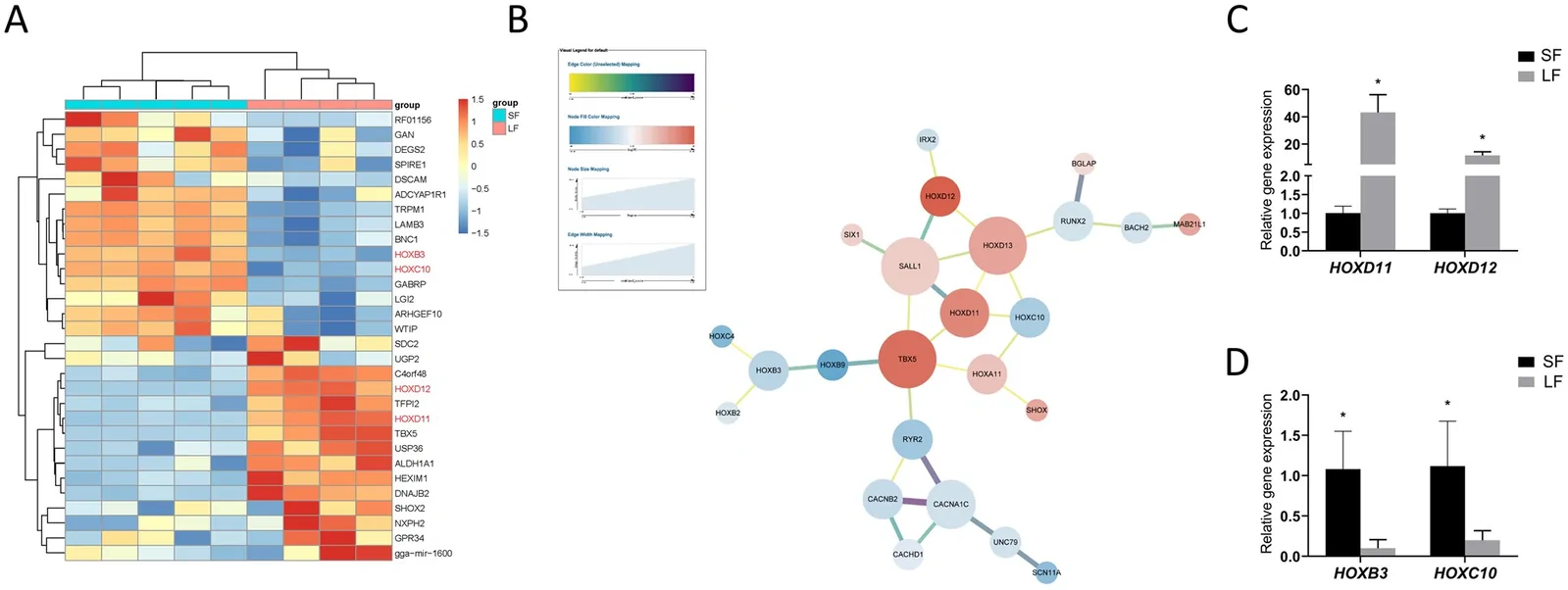
Protein interaction network construction and validation of DEGs in yellow-feathered chickens. A) Heatmap of top 15 upregulated and downregulated genes based on *P*-value screening. B) Protein–protein interaction network of differentially expressed HOX proteins and their directly interacting proteins. Red and blue nodes indicate upregulation and downregulation, respectively, in the LF group relative to the SF group. Node size represents degree, whereas edge width represent the STRING combined interaction score. C) The mRNA expression of upregulated genes was measured using qRT-PCR (*n* = 5). D) The mRNA expression of downregulated genes was measured using qRT-PCR (*n* = 5). Data are shown as mean ± SD, * indicates *P *< 0.05.

To validate the RNA-seq results and identify genes involved in regulating chicken feather follicles, we conducted qRT-PCR analysis of the selected differentially expressed *HOX* genes using five biological samples per group (Figure 5C and D). The log₂ fold change measured by qRT-PCR showed a strong positive correlation with those obtained by RNA-seq for the four validated *HOX* genes, supporting the consistency of the overall expression trends between the two methods (Figure S2, see online supplementary material for a color version of this figure).

### HOXB3 gene polymorphisms and identification of valuable SNPs associated with chicken feather follicle size trait

*HOXB3* was significantly downregulated in the LF group relative to the SF group and exhibited a relatively large magnitude of differential expression among the differentially expressed *HOX* genes. It also exhibited relatively high connectivity with other HOX proteins in the predicted protein–protein interaction network. Together with its relatively high expression in skin tissue, these findings suggest that *HOXB3* may be associated with the SF phenotype and supported its selection for subsequent polymorphism and association analyses. We then analyzed its mRNA expression levels in various tissues using qPCR in four 100-d-old yellow-feathered chickens. The results showed that *HOXB3* expression was highest in skin tissue, suggesting that *HOXB3* plays a pivotal role in skin and feather follicle development (Figure 6). This finding is also consistent with the transcriptome results across different tissues. We then screened the *HOXB3* gene as a candidate to identify SNPs markers associated with chicken feather follicle size traits. Primers targeting the *HOXB3* gene sequence were designed and synthesized for PCR amplification. Sanger sequencing was performed on PCR products from 332 randomly selected yellow-feathered chickens, followed by genotyping (Figure S3, see online supplementary material for a color version of this figure). As shown in Table 3, a total of 14 SNPs were detected in the *HOXB3* gene. Of these, one SNP is located in the 5’ untranslated region (UTR), eight in the intron region, and five in the exon region. All SNPs in exon 5/coding sequences are synonymous mutations, meaning they do not alter the amino acid encoded.

**Figure 6:**
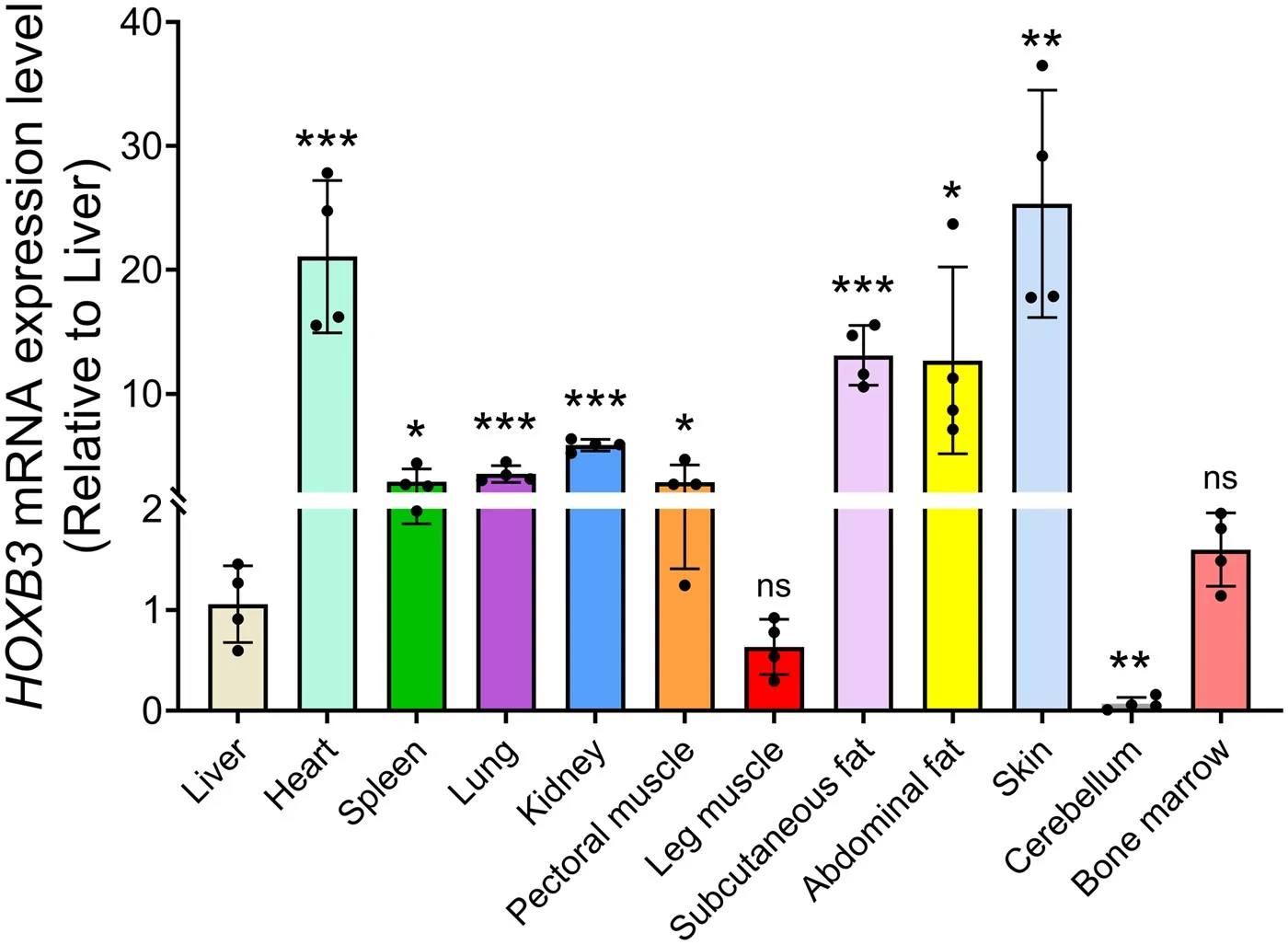
The expression of *HOXB3* mRNA in different tissues detected by qRT-PCR (*n* = 4 samples of each tissue). *, **, *** indicate *P *< 0.05, *P *< 0.01, *P *< 0.001, respectively; and ns denotes non-significance difference at *P *> 0.05.

**Table 3:** SNPs detected in the *HOXB3* gene in yellow-feathered chickens (*n* = 332).

SNPs^1^	Chromosome position	Location	Amino acid change
**rs735806371 (C > A)**	27: 6268764	Intron4	Non-transcription sequence
**rs315593398 (T > C)**	27: 6268799	Intron4	Non-transcription sequence
**rs14732927 (C > A)**	27: 6268888	Intron4	Non-transcription sequence
**rs313555228 (C > G)**	27: 6268958	Intron4	Non-transcription sequence
**rs315560518 (A > T)**	27: 6268995	Intron4	Non-transcription sequence
**rs14732926 (G > A)**	27: 6268997	Intron4	Non-transcription sequence
**rs14732925 (A > G)**	27: 6269439	5’ UTR^2^	Non-coding sequence
**rs14732924 (G > A)**	27: 6269490	Exon5	Synonymous mutation
**rs14732923 (C > T)**	27: 6269544	Exon5	Synonymous mutation
**rs736476363 (C > T)**	27: 6269616	Exon5	Synonymous mutation
**rs14732922 (C > T)**	27: 6269736	Exon5	Synonymous mutation
**rs740772872 (G > A)**	27: 6269865	Exon5	Synonymous mutation
**rs316447826 (A > G)**	27: 6269901	Intron5	Non-transcription sequence
**rs312469598 (A > G)**	27: 6269918	Intron5	Non-transcription sequence

^1^ SNPs were detected by Sanger sequencing in 332 randomly selected yellow-feathered chickens.

^2^ UTR = untranslated region.

Next, we calculated the allele and genotype frequencies of the 14 SNPs, as shown in Table 4. Two genotypes were detected at three of the 14 mutation sites, while the remaining 11 sites exhibited three genotypes, possibly due to population size. Except for three SNPs (rs14732927, rs313555228, and rs14732926), which had higher mutant allele frequencies compared to the reference allele, the other SNPs exhibited higher reference allele frequencies. Chi-square tests were conducted to assess the Hardy–Weinberg equilibrium for each SNP. The analysis showed that all SNPs conformed to the Hardy–Weinberg equilibrium. To place these results in a broader population context, publicly available genotype and allele frequency data were retrieved from Ensembl/EVA study PRJEB44919 for five SNPs: rs315593398, rs14732927, rs313555228, rs315560518, and rs14732926. Although the allele frequencies varied among our yellow-feathered chicken population and the publicly available Chinese and Korean chicken populations, the same major allele was observed at all five loci (Table S6). We also calculated the genetic diversity indices of all SNPs, with the results for the *HOXB3* SNPs presented in Table 5. Regarding observed and expected heterozygosity, SNPs rs735806371, rs315593398, rs315560518, rs14732925, and rs740772872 had higher observed heterozygosity than expected. In contrast, SNPs rs313555228, rs14732926, rs14732924, rs736476363, and rs312469598 exhibited lower observed heterozygosity than expected. For the remaining SNPs, observed and expected heterozygosity were approximately equal. Additionally, all SNPs exhibited low to medium polymorphism levels based on PIC. The effective number of alleles ranged from 1.111 to 1.984, with rs313555228 showing the highest value and rs315560518 the lowest. Notably, both rs313555228 and rs315560518 are located in intronic regions.

**Table 4:** The allele information of SNPs in the *HOXB3* gene in yellow-feathered chickens.

SNPs	*N*	Genotypes of alleles			**Genotype frequency** ^1^			Allele frequency		HWE *P*-value^2^
**rs735806371**	332	CC (*n* = 295)	CA (*n* = 37)		0.889	0.111		P(C) = 0.944	P(A) = 0.056	0.561
**rs315593398**	332	TT (*n* = 233)	TC (*n* = 93)	CC (*n* = 6)	0.702	0.280	0.018	P(T) = 0.842	P(C) = 0.158	0.637
**rs14732927**	332	CC (*n* = 58)	CA (*n* = 162)	AA (*n* = 112)	0.175	0.488	0.337	P(C) = 0.419	P(A) = 0.581	1.000
**rs313555228**	332	CC (*n* = 74)	CG (*n* = 154)	GG (*n* = 104)	0.223	0.464	0.313	P(C) = 0.455	P(G) = 0.545	0.500
**rs315560518**	332	AA (*n* = 297)	AT (*n* = 35)		0.895	0.105		P(A) = 0.947	P(T) = 0.053	0.598
**rs14732926**	332	GG (*n* = 8)	GA (*n* = 80)	AA (*n* = 244)	0.024	0.241	0.735	P(G) = 0.145	P(A) = 0.855	0.895
**rs14732925**	332	AA (*n* = 213)	AG (*n* = 108)	GG (*n* = 11)	0.642	0.325	0.033	P(A) = 0.804	P(G) = 0.196	0.834
**rs14732924**	332	GG (*n* = 239)	GA (*n* = 83)	AA (*n* = 10)	0.720	0.250	0.030	P(G) = 0.845	P(A) = 0.155	0.701
**rs14732923**	332	CC (*n* = 236)	CT (*n* = 88)	TT (*n* = 8)	0.711	0.265	0.024	P(C) = 0.843	P(T) = 0.157	0.998
**rs736476363**	332	CC (*n* = 191)	CT (*n* = 120)	TT (*n* = 21)	0.575	0.361	0.063	P(C) = 0.756	P(T) = 0.244	0.934
**rs14732922**	332	CC (*n* = 207)	CT (*n* = 110)	TT (*n* = 15)	0.623	0.331	0.045	P(C) = 0.789	P(T) = 0.211	0.997
**rs740772872**	332	GG (*n* = 279)	GA (*n* = 53)		0.840	0.160		P(G) = 0.920	P(A) = 0.080	0.287
**rs316447826**	332	AA (*n* = 130)	AG (*n* = 156)	GG (*n* = 46)	0.392	0.470	0.139	P(A) = 0.627	P(G) = 0.373	0.997
**rs312469598**	332	AA (*n* = 240)	AG (*n* = 82)	GG (*n* = 10)	0.723	0.247	0.030	P(A) = 0.846	P(G) = 0.154	0.658

*N* = number of chickens.

1 Genotype frequency: The genotype order of genotypic frequency is the same as in the “Genotypes of Alleles” column.

2 HWE *P*-values were obtained using chi-square tests comparing the observed and expected genotype frequencies for each SNP. *P *> 0.05 indicates no significant deviation from Hardy–Weinberg equilibrium.

**Table 5:** The genetic diversity indices of SNPs in the *HOXB3* gene in yellow-feathered chickens (*n* = 332).

SNPs	Ho	He	**PIC** ^1^	Ne
**rs735806371**	0.111	0.105	0.100	1.118
**rs315593398**	0.280	0.266	0.231	1.363
**rs14732927**	0.488	0.487	0.368	1.948
**rs313555228**	0.464	0.496	0.373	1.984
**rs315560518**	0.105	0.100	0.095	1.111
**rs14732926**	0.241	0.247	0.217	1.329
**rs14732925**	0.325	0.315	0.265	1.460
**rs14732924**	0.250	0.262	0.228	1.355
**rs14732923**	0.265	0.264	0.229	1.359
**rs736476363**	0.361	0.369	0.301	1.585
**rs14732922**	0.331	0.333	0.277	1.499
**rs740772872**	0.160	0.147	0.136	1.172
**rs316447826**	0.470	0.468	0.358	1.880
**rs312469598**	0.247	0.260	0.226	1.351

Ho = observed heterozygosity; He = expected heterozygosity; Ne = effective number of alleles.

1 PIC > 0.5 means high polymorphism, 0.25 < PIC < 0.5 means intermediate polymorphism, PIC < 0.25 means low polymorphism.

Finally, we performed association analyses between the 14 SNPs and back feather follicle size (BFFS) in chickens. To evaluate whether the randomly selected samples accurately represent the overall population, we generated BFFS distribution histograms for both the entire population and the sequencing subset. The mean BFFS values were 199.23 ± 34.67 for the total population and 198.83 ± 34.32 for the test samples. The density curves of the two groups substantially overlapped, and their mean values were very close (Figure S4, see online supplementary material for a color version of this figure). These results suggest that the test samples were broadly representative of the overall population with respect to BFFS. Among these, three SNPs (rs14732924, rs736476363, and rs14732922) were significantly associated with BFFS, as shown in Table 6. Specifically, BFFS were significantly higher in the GG genotype of rs14732924 compared to the AA genotype. Similarly, BFFS were significantly higher in the TT genotype of rs736476363 than in the CC and CT genotypes. BFFS were significantly higher in the CC genotype than in the TT genotype for rs14732922.

**Table 6:** Association analysis of SNPs in *HOXB3* gene with BFFS in yellow-feathered chickens (*n* = 332).

SNPs	Genotype	Mean ± SD^1^
**rs14732924**	GG (*n* = 239)	204.60 ± 5.48^a^
	GA (*n* = 83)	198.35 ± 6.71^ab^
	AA (*n* = 10)	176.52 ± 13.61^b^
**rs736476363**	CC (*n* = 191)	203.75 ± 6.29^a^
	CT (*n* = 120)	203.19 ± 4.88^a^
	TT (*n* = 21)	232.83 ± 8.94^b^
**rs14732922**	CC (*n* = 207)	211.38 ± 7.51^a^
	CT (*n* = 110)	201.76 ± 5.63^ab^
	TT (*n* = 15)	192.32 ± 9.56^b^

1 Different lowercase letters indicate significant differences in means (*P *< 0.05), and the same letters indicate insignificant differences in means (*P *> 0.05).

## Discussion

Feather follicle traits are important components of chicken carcass appearance. The main follicle traits include density, size, and diameter. Follicle density is commonly measured by counting the number of follicles per unit area (Ji et al. 2021). The size and diameter of follicles are traditionally assessed using hematoxylin and eosin-stained sections (Chen et al. 2023). However, histological analysis is costly and time-consuming and is therefore not well suited to large-scale phenotyping. In the present study, standardized skin microscopy combined with ImageJ converted visual differences in feather follicle size into quantitative pixel-based measurements. This approach provided a practical method for the relative comparison of follicle size under standardized imaging conditions, although further validation against histological measurements is needed. Because feather follicles support feather growth and development, follicle and feather traits may be biologically related. A previous study used rachis width or cross-sectional area as an indirect indicator of feather follicle size and found that rachis cross-sectional area was positively correlated with feather length and mass (Jenni et al. 2020). In our study, feather follicle size also showed significant positive phenotypic correlations with feather length and mass, whereas feather follicle density was negatively correlated with several feather traits. Genetic relationships have also been reported among feather traits. In ostriches, average feather length and width showed moderate to high genetic correlations with feather weight (Brand and Cloete 2015). Similarly, feather mass showed moderate to high genetic correlations with feather length and calamus diameter, particularly for flight feathers. The heritability of feather traits has been evaluated in several avian species, but estimates vary among species and body regions. For example, the heritability of flight feather mass was estimated to be 0.65 in the migratory bird Ficedula hypoleuca (De La Hera et al. 2013). In pheasants, the heritability estimates for tail feather length and primary flight feather length were 0.30 and 0.14, respectively (Rizzi et al. 1994). In the present study, the heritability estimates for flight feather length and mass were 0.45 and 0.44, respectively, whereas the heritability of back contour feather length was only 0.10. Differences among studies may reflect variation in species, genetic background, body region, age, and measurement methods. These results suggest that flight feather length and mass may have greater potential for genetic improvement than back contour feather length.

Our findings revealed that male chickens exhibited significantly higher feather trait values compared to females, indicating a sex disparity in feather growth. Additionally, a significant sex difference was observed in BFFS among follicle traits, while no significant differences were found in BFFSI or DFF. Previous studies have implicated estrogen and testosterone in the sexual dimorphism of chicken plumage (Wilson et al. 1987; Widelitz et al. 2019), suggesting that hormonal regulation may partly explain the sex-associated differences in feather and feather follicle traits observed in the present study. We then observed a variation coefficient of approximately 13% for feather follicles at the same site within an individual, highlighting the challenges of accurately determining feather follicle size. Thus, identifying an indirect selection indicator emerged as a potential solution. Indirect selection involves selecting one trait associated with another when the latter cannot be directly selected or when direct selection is less effective. Previous research by Whitehead and Griffin (1986) identified a strong positive correlation between plasma VLDL concentrations and body fat mass in broilers, using this indicator to breed a lean chicken strain (Whitehead and Griffin 1986). In our study, BFFS exhibited a strong negative genetic correlation with WCF and LCF, potentially due to the role of feather follicles in providing nutrients for feather growth (Lin et al. 2013). Additionally, converting BFFS into a feather follicle size index revealed a strong negative genetic correlation with LCF. Therefore, LCF can serve as an indirect selection indicator for feather follicle size selection. Studies have also identified significant correlations among most feather traits, consistent with findings from previous research (Jenni et al. 2020).

Feather follicle traits are complex quantitative traits regulated by multiple genes. Their formation and development involve a complex physiological process, predominantly regulated by genetic factors. To investigate the molecular regulatory mechanisms of feather follicle size, we performed transcriptome sequencing on secondary flight feather follicle tissues with large follicles and dorsopelvic tract tissues with small follicles. We identified 1,273 DEGs in this analysis. These DEGs were further analyzed for their functions and pathways using GO enrichment and KEGG pathway analyses. GO analysis revealed that the DEGs were primarily involved in tissue and organ development, highlighting the complexity of feather follicle formation. Additionally, most DEGs were located at the cell membrane, emphasizing the importance of cell-to-cell interactions during feather follicle development, particularly between epidermal and dermal cells (Shin et al. 2010). KEGG pathway analysis revealed that the DEGs were mainly associated with the Wnt, MAPK, calcium, and TGF-β signaling pathways, consistent with previous studies on chicken feather follicle development (Gong et al. 2018). The Wnt signaling pathway is pivotal in the morphogenesis and development of feather follicles in avian skin. The Wnt signaling pathway primarily operates through two pathways: the canonical Wnt/β-catenin pathway and the non-canonical pathway. In the canonical pathway, Wnt proteins activate the Frizzled receptor family and transmit signals via low-density lipoprotein receptor-related proteins 5/6 (LRP5/6). This process leads to β-catenin accumulation and nuclear translocation, ultimately activating genes that regulate cell proliferation and migration (Chen et al. 2020). In the non-canonical pathway, Wnt proteins regulates feather follicle morphogenesis by modulating cell polarity and calcium signaling (Chen et al. 2020). During different stages of feather development, specific Wnt proteins such as Wnt1 and Wnt3a contribute to feather trajectory formation, while Wnt7a and Wnt11 are essential for the localization and boundary formation of feather buds (Ji et al. 2023). Bone morphogenetic proteins (BMPs) in the TGF-β superfamily suppress feather follicle formation. BMPs regulate the location and density of feather buds by producing inhibitory signals in specific skin regions (Noramly et al. 1999). These inhibitory effects are comparable to the activation of the FGF signaling pathway, jointly influencing feather follicle formation and arrangement (Ho et al. 2019). The MAPK signaling pathway is notably associated with feather development, with studies suggesting that FGF inhibits feather growth through the MAPK pathway (Chen et al. 2016). This pathway also regulates epithelial cell proliferation, junction remodeling, and bud formation, contributing to normal hair morphogenesis (Rishikaysh et al. 2014). Interestingly, we found that the DEGs were enriched in the melanogenesis process, suggesting variations in melanin deposition in feather follicles of varying sizes and densities. This observation aligns with findings from a previous study (Ji et al. 2021). Given that feather color is closely associated with the number and distribution of melanocytes in feather follicles (Hellström et al. 2010), this could explain why the tail vertebra feathers in chickens appear darker than the secondary wing feathers. These findings further underscore the critical role of regulatory pathways in the formation and development of feather follicles.

Further analysis identified several *HOX* family genes, including *HOXC10*, *HOXD11*, *HOXB3*, and *HOXD12*, among the top-ranked DEGs. Previous studies have shown that *HOX* genes are involved in embryonic development, epidermal differentiation, and feather follicle morphogenesis (Awgulewitsch 2003; Yang et al. 2010; Montavon and Soshnikova 2014). *HOXC* genes have also been implicated in feather follicle regeneration through the Wnt signaling pathway (Yu et al. 2018), and *HOXC12* and *HOXC13* show stage-specific expression during the feather follicle cycle (Shang et al. 2002). Consistent with these findings, several *HOX* family members were differentially expressed between the LF and SF groups in the present study. Members of the *HOXB* and *HOXC* families were predominantly downregulated, whereas members of the *HOXA* and *HOXD* families were predominantly upregulated in the LF group relative to the SF group. *TBX5* and *SALL1* were also connected with several HOX proteins in the predicted protein–protein interaction network. Previous studies have shown that *SALL1* is involved in chicken wing-bud development, whereas ectopic expression of *TBX5* in hindlimb buds can contribute to feathered-leg formation (Welten et al. 2011; Li et al. 2020). These observations suggest that the proteins identified in the network may be related to the regional development of skin and feather structures, although their regulatory relationships require further validation.

Previous genetic studies in chickens further support the involvement of *HOXB* genes in feather development. A copy-number variation encompassing *HOXB7* and *HOXB8* was associated with the beard phenotype in chickens, and these proteins showed strong predicted interactions with other *HOXB* family members (Yang et al. 2020). In addition, ectopic upregulation of *HOXB8* in mandibular skin altered skin morphology and the expression of genes related to keratin synthesis and feather follicle development (Zheng et al. 2023). In the present study, *HOXB3* was downregulated in the LF group relative to the SF group, showed a relatively large magnitude of differential expression, and exhibited relatively high connectivity with other HOX proteins in the predicted network. These findings supported its selection as a candidate gene for polymorphism analysis. Three *HOXB3* SNPs, rs14732924, rs736476363, and rs14732922, were significantly associated with BFFS. This result extends previous evidence linking *HOXB* genes to chicken feather traits and suggests that *HOXB3* may also contribute to variation in feather follicle size. However, further functional studies are needed to determine whether these SNPs directly affect *HOXB3* expression or feather follicle development.

## Conclusion

This study characterized the phenotypic variation, genetic parameters, and molecular features associated with feather follicle size in yellow-feathered chickens. The genetic relationship between LCF and BFFS suggests that LCF may serve as a practical indirect selection indicator for feather follicle size. Transcriptomic and association analyses further identified Wnt, MAPK, calcium, and TGF-β signaling pathways, as well as several *HOX* genes, as potential contributors to feather follicle development. In particular, *HOXB3* and its associated SNPs represent promising candidates for genetic improvement of feather follicle traits. However, the effects of these candidate genes and SNPs require further validation in larger independent populations and through functional experiments. Overall, these findings provide a basis for understanding the genetic regulation of feather follicle development and for improving feather follicle traits in yellow-feathered chickens.

## Supplementary Material

skag291_Supplementary_Data

## Data Availability

The raw sequence data reported in this paper have been deposited in the Genome Sequence Archive in National Genomics Data Center, China National Center for Bioinformation/Beijing Institute of Genomics, Chinese Academy of Sciences (GSA: CRA020060) that are publicly accessible at https://ngdc.cncb.ac.cn/gsa.

## Abbreviations

BFFS: feather follicle size on back
BFFSI: feather follicle size index on back
BMPs: bone morphogenetic proteins
BW: body weight
*CDK1*: cyclin-dependent kinase 1
DCFC: diameter of contour feather calamus on back
DEGs: differentially expressed genes
DFF: density of feather follicle
DFFC: diameter of flight feather calamus
*DHRS12*: dehydrogenase/reductase 12
*DNAJC15*: DnaJ heat shock protein family (Hsp40) member C15
ECM: extracellular matrix
FGF: fibroblast growth factor
*FGF9*: fibroblast growth factor 9
*FOXM1*: forkhead box M1
FPKM: fragments per kilobase of transcript per million mapped reads
*GAPDH*: glyceraldehyde-3-phosphate dehydrogenase
GO: Gene Ontology
*GTSE1*: G2 and S phase expressed 1
GWAS: genome-wide association studies
He: expected heterozygosity
Ho: observed heterozygosity
*HOX*: homeobox
*HOXA7*: homeobox A7
*HOXA11*: homeobox A11
*HOXB3*: homeobox B3
*HOXB4*: homeobox B4
*HOXB6*: homeobox B6
*HOXC6*: homeobox C6
*HOXC8*: homeobox C8
*HOXC10*: homeobox C10
*HOXC12*: homeobox C12
*HOXC13*: homeobox C13
*HOXD4*: homeobox D4
*HOXD11*: homeobox D11
*HOXD12*: homeobox D12
*HOXD13*: homeobox D13
*IGF1*: insulin-like growth factor 1
KEGG: Kyoto encyclopedia of genes and genomes
LCF: length of contour feather on back
LCFC: length of contour feather calamus on back
LF: large feather follicles
LFF: length of flight feather
LFFC: length of flight feather calamus
*LRP5/6*: lipoprotein receptor-related proteins 5/6
MAPK: mitogen-activated protein kinase
*MELK*: maternal embryonic leucine zipper kinase
*MLNR*: motilin receptor
Ne: effective number of alleles
OD: optical density
PCR: polymerase chain reaction
PIC: polymorphism information content
*RB1*: retinoblastoma 1
*SALL1*: spalt like transcription factor 1
SF: small feather follicles
SNP: single nucleotide polymorphism
*SUCLA2*: succinate-CoA ligase ADP-forming subunit beta
*TBX5*: T-Box 5
TGF-β: transforming growth factor beta
UTR: untranslated region
VLDL: very-low-density lipoprotein
WCF: weight of contour feather on back
WFF: weight of flight feather
Wnt: wingless-related integration site
Wnt1: wingless-related integration site 1
Wnt11: wingless-related integration site 11
Wnt3a: wingless-related integration site 3a
Wnt7a: wingless-related integration site 7a
