# Supplementary material for: Identification of candidate genes associated with feather follicle traits in chickens
Source: J Anim Sci. 2026 Nov 5;104:skag291. doi: 10.1093/jas/skag291 (PMC13765533; doi:10.1093/jas/skag291)
Supplement: Supplementary file 1 [file skag291_supplementary_data.zip › Supplementary Materials.docx]

Supplementary materials-Tables

**Table S1.** The nutritional level at different feeding stages (as-fed basis).

| Feeding Age, day | ME^1^, MJ/kg | Crude Protein, % | Crude Fiber, % | Ash, % | Calcium, % | Total Phosphorus, % | Sodium Chloride, % | Lysine, % | Methionine, % |
| --- | --- | --- | --- | --- | --- | --- | --- | --- | --- |
| 1-35 | 12.47 | 19.50 | 5.00 | 8.00 | 0.75-1.10 | 0.40 | 0.25-0.80 | 1.00 | 0.40 |
| 36-45 | 12.69 | 17.75 | 5.00 | 7.50 | 0.60-1.10 | 0.40 | 0.25-0.80 | 0.85-1.00 | 0.30-0.40 |
| 46-100 | 12.91 | 16.00 | 5.00 | 7.00 | 0.60-0.90 | 0.40 | 0.25-0.80 | 0.85 | 0.30 |

^1^ME: Metabolizable Energy.

**Table S2.** Primer sequences in PCR.

| Gene | Primer sequence (5’-3’) | Temperature, °C | Size, bp |
| --- | --- | --- | --- |
| *HOXB3* | F- AAACAGGCCAAAGGAAGCAC | 57 | 1441 |
|  | R- CAGTATCTGCATCCTCGCTGT |  |  |

**Table S3.** Primer sequences in reverse-transcription quantitative PCR.

| Gene | Primer sequence (5’ - 3’) | Temperature, °C | Size, bp |
| --- | --- | --- | --- |
| *HOXB3* | F- AGAAAAGCAGCTCCCCTAGC | 58  60  60  58 | 143  81  148  111 |
| *HOXC10*  *HOXD11*  *HOXD12* | R- CAGGTAGCGGTTGAAGTGGA  F- ACCCAAGGAACGAGCATCTG  R- AACTCACTTTAGCCACCGGG  F- GTTCAGCAGTTCCTCAGCGA  R- CGGTCAGTGAGGTTGAGCAT  F- GGCTGGTCCCTTCATGTGTT  R- TTTGGTAACCGCTTTGTGCG |  |  |
| *GAPDH* | F- AGAACATCATCCCAGCGT | 56 | 182 |
|  | R- AGCCTTCACTACCCTCTTG |  |  |

**Table S4.** The details of genomic alignment of individual sample reads.

| Sample | Total Reads | Mapped reads | Overall mapping rate | Unique mapping rate | Number of expressed genes |
| --- | --- | --- | --- | --- | --- |
| LF1 | 44232660 | 40989443 | 92.67% | 82.86% | 16818 |
| LF2 | 43586384 | 39859602 | 91.45% | 87.66% | 17049 |
| LF3 | 41715762 | 37947415 | 90.97% | 88.08% | 16625 |
| LF4 | 40936206 | 37808139 | 92.36% | 90.29% | 17186 |
| SF1 | 42814894 | 39454142 | 92.15% | 89.45% | 17447 |
| SF2 | 45450472 | 41991039 | 92.39% | 90.05% | 16523 |
| SF3 | 43581320 | 40041748 | 91.88% | 85.73% | 17581 |
| SF4 | 40704554 | 37754096 | 92.75% | 88.62% | 17525 |
| SF5 | 43964828 | 40983262 | 93.22% | 89.40% | 17744 |

Expressed genes were defined as genes with FPKM > 0.1.

**Table S5.** Top-ranked differentially expressed genes between the LF and SF groups based on adjusted *P*-values.

| Gene | LF (Average Expression) | SF (Average Expression) | Adjusted *P*-value | Code Capacity | Gene Family |
| --- | --- | --- | --- | --- | --- |
| *HOXC10* | 24.39 | 577.32 | 9.36E-28 | Protein | *Homeobox* |
| *HOXD11* | 265.55 | 1.26 | 6.75E-27 | Protein | *Homeobox* |
| *HEXIM1* | 6281.75 | 3306.8 | 6.75E-27 | Protein | *HEXIM* |
| *C4orf48* | 593.49 | 112.16 | 2.10E-26 | Protein | *-* |
| *HOXB3* | 17.24 | 204.87 | 3.90E-23 | Protein | *Homeobox* |
| *HOXD12* | 208.73 | 0.15 | 9.75E-23 | Protein | *Homeobox* |

**Table S6.** Genotype and allele frequencies of *HOXB3* SNPs in publicly available chicken populations

| SNPs | Population | N^1^ | Genotype (count) | | | Genotype frequency | | | Allele frequency | | Data source |
| --- | --- | --- | --- | --- | --- | --- | --- | --- | --- | --- | --- |
| rs315593398 | China | 60 | TT (39) | TC (16) | CC (5) | 0.650 | 0.267 | 0.083 | P(T) = 0.783 | P(C) = 0.217 | Ensembl/EVA PRJEB44919 |
|  | Korea | 110 | TT (64) | TC (30) | CC (16) | 0.582 | 0.273 | 0.145 | P(T) = 0.718 | P(C) = 0.282 |  |
| rs14732927 | China | 55 | AA (35) | AC (20) |  | 0.636 | 0.364 |  | P(A) = 0.818 | P(C) = 0.182 |  |
|  | Korea | 108 | AA (78) | AC (30) |  | 0.722 | 0.278 |  | P(A) = 0.861 | P(C) = 0.139 |  |
| rs313555228 | China | 62 | GG (41) | GC (21) |  | 0.661 | 0.339 |  | P(G) = 0.831 | P(C) = 0.169 |  |
|  | Korea | 113 | GG (81) | GC (32) |  | 0.717 | 0.283 |  | P(G) = 0.858 | P(C) = 0.142 |  |
| rs315560518 | China | 62 | AA (46) | AT (14) | TT (2) | 0.742 | 0.226 | 0.032 | P(A) = 0.855 | P(T) = 0.145 |  |
|  | Korea | 114 | AA (97) | AT (15) | TT (2) | 0.851 | 0.132 | 0.018 | P(A) = 0.917 | P(T) = 0.083 |  |
| rs14732926 | China | 61 | AA (50) | AG (11) |  | 0.820 | 0.180 |  | P(A) = 0.910 | P(G) = 0.090 |  |
|  | Korea | 113 | AA (91) | AG (22) |  | 0.805 | 0.195 |  | P(A) = 0.903 | P(G) = 0.097 |  |

¹N represents the number of chickens and was calculated from the reported genotype counts.

Genotype and allele frequencies were obtained from Ensembl population genetics data derived from EVA study PRJEB44919.
